# Eliminating perinatal transmission of hepatitis B virus: it is time for action

**DOI:** 10.1002/jia2.26337

**Published:** 2024-07-25

**Authors:** Rania A. Tohme, Su Wang, Benjamin Cowie, Sandra Dudareva, Carolyn Wester

**Affiliations:** ^1^ Division of Viral Hepatitis U.S. Centers for Disease Control and Prevention Atlanta Georgia USA; ^2^ Cooperman Barnabas Medical Center and Hepatitis B Foundation Livingston New Jersey USA; ^3^ WHO Collaborating Centre for Viral Hepatitis The Peter Doherty Institute for Infection and Immunity Melbourne Victoria Australia; ^4^ Department of Infectious Disease Epidemiology Robert Koch Institute Berlin Germany

1

Chronic hepatitis B virus (HBV) infection is a leading cause of liver cirrhosis and liver cancer causing 1.1 million deaths globally in 2022 [1]. In 2022, an estimated 254 million persons were living with chronic HBV infection. Globally, HBV is mainly acquired through mother‐to‐child transmission (MTCT) at birth (vertical or perinatal transmission), and during early childhood (horizontal transmission). Up to 90% of newborns who acquire HBV through MTCT will develop chronic hepatitis B compared to 30%–50% of children infected between the ages of 1–5 years, while <5% of those infected in adulthood develop chronic hepatitis B [2]. The hepatitis B vaccine is >90% effective at preventing infections and is given as a series starting with a dose of monovalent vaccine within 24 hours of birth (hepatitis B‐birth dose [hepB‐BD]) (70%–95% effective in preventing perinatal HBV infection), followed by two or three additional doses during infancy [2].

Elimination targets for MTCT of HBV include achieving ≤0.1% prevalence of hepatitis B surface antigen (HBsAg) in children ≤5 years of age, and ≥90% coverage with timely HepB‐BD and three doses of hepatitis B vaccine (HepB3) [3]. In addition, countries that provide selective HepB‐BD (e.g. only to infants with known exposure) need to screen ≥90% of pregnant women for hepatitis B and treat ≥90% of those eligible [3]. Prevention of HBV infection in infancy and childhood through vaccination and treatment of pregnant women would be the most impactful interventions to reduce the prevalence of chronic hepatitis B in the population.

An analysis of the impact of childhood vaccination in 98 low‐ and middle‐income countries showed that hepatitis B vaccination will have prevented 38 million (range: 25–52 million) deaths over the lifetime of those born from 2000 to 2030, which was second only to measles vaccine [4]. Yet, despite the availability of safe and effective hepatitis B vaccines since 1982, coverage with timely hepB‐BD has been suboptimal in most regions (Figure 1). By 2023, 140 of 195 (72%) countries have introduced either universal or selective HepB‐BD, with 115 (59%) countries providing HepB‐BD to all newborns [5]. In 2022, almost 5.6 million children aged ≤5 years were living with HBV infection [6]. In the World Health Organization (WHO) African region where the burden of HBV infection in children is the highest, only 16 of 47 (34%) countries have introduced HepB‐BD mainly due to lack of financial support from Gavi, the Vaccine Alliance [7]. In 2020, Gavi approved funding for HepB‐BD introduction; however, this was put on hold due to the COVID‐19 pandemic. In June 2024, Gavi launched official funding support for eligible countries for HepB‐BD introduction [8]. Countries must now take urgent action to introduce HepB‐BD, including submitting applications for Gavi funding.

**Figure 1:**
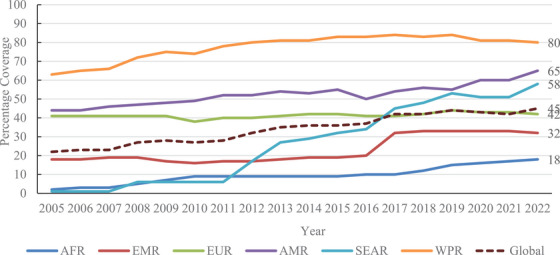
Timely hepatitis B birth dose vaccination coverage by WHO region, 2005–2022. AFR, African region; AMR, region of the Americas; EMR, Eastern Mediterranean region; EUR, European region; SEAR, South‐East Asia region; WPR, Western Pacific region. *Source*: https://immunizationdata.who.int/global/wiise‐detail‐page/hepatitis‐b‐vaccination‐coverage?CODE=Global&GROUP=WHO_REGIONS&ANTIGEN=HEPB_BD&YEAR=.

Strategies to increase timely HepB‐BD coverage for both in‐facility and outside‐of‐health facility births also need to be implemented [9]. Educating healthcare workers, encouraging women to deliver in health facilities, providing the vaccine through the maternal child health programme in the delivery wards rather than immunization clinics and ensuring vaccine availability in delivery wards have been shown to improve timely HepB‐BD coverage for health facility births [9]. To reach home births, educating pregnant women and community health workers on the importance of timely HepB‐BD, leveraging community health workers to identify all pregnant women and notify health facilities of recent births, integrating administration of HepB‐BD during post‐natal care visits and using compact, prefilled auto‐disable devices that require less training to administer have all been shown to increase coverage with timely HepB‐BD [9].

Furthermore, innovations in vaccine preparation and administration, such as the hepatitis B vaccine microneedle patch (MNP), have been shown to elicit robust immunologic responses in animal studies [10]. Recently, a phase 1/2 clinical trial for a measles and rubella vaccine (MRV)‐MNP among children and adults in the Gambia was shown to be safe and immunogenic, supporting the accelerated development of MRV‐MNP [11]. Similar innovative strategies for the hepatitis B vaccine need to be prioritized to close the gap in timely HepB‐BD coverage, especially in high‐prevalence settings.

While vaccination is the necessary starting point for the prevention of MTCT of HBV, it is not sufficient to accelerate the elimination of hepatitis B. Screening of pregnant women for HBV infection and providing free antiviral treatment for those eligible are additionally needed to ensure maximal prevention of perinatal infections and prevent progression of liver disease in women [12]. Integration of elimination of MTCT (eMTCT) of HBV with frequently already implemented HIV and syphilis services can be cost‐effective and feasible, as has been shown in Cambodia and Vietnam [13, 14]. For example, existing HIV platforms to prevent MTCT of HIV in sub‐Saharan Africa funded through the President's Emergency Plan for AIDS Relief (PEPFAR) provide an opportunity to integrate triple eMTCT of HIV, syphilis and HBV. Therefore, it is time to promote concurrent screening and linkage to care for all three infections during antenatal care.

Furthermore, the WHO's newly simplified hepatitis B management guidelines present an opportunity to scale up treatment for hepatitis B among pregnant women in settings where molecular testing for HBV DNA is not available [12]. In 2022, only 3% of pregnant women with high viral load received antiviral treatment [6]. While simplification of the hepatitis B treatment guidelines might increase the proportion of pregnant women treated, availability of free treatment and follow‐up of pregnant persons with HBV infection are needed to assess long‐term treatment eligibility for their own health [12]. Incorporating screening and treatment data on hepatitis B into existing data systems platforms is also essential to track the percentage of persons diagnosed and treated and measure progress towards hepatitis B elimination.

In conclusion, the tools are available to eliminate perinatal transmission of HBV. The WHO has developed a framework to guide the implementation of person‐centred and integrated interventions to scale‐up triple eMTCT [15]. It is time for joint and coordinated action to ensure integrated approaches to scale up eMTCT under the framework of universal health coverage to give the next generation a hepatitis B‐free future and achieve elimination by 2030.

## COMPETING INTERESTS

SW received research funding for her institution from Gilead Sciences. All other authors do not have any conflicts of interest to declare.

## AUTHORS’ CONTRIBUTIONS

RAT led the write‐up of the manuscript. SW, BC, SD and CW contributed ideas to the manuscript, reviewed it and agreed with its final version. The findings and conclusions in this manuscript are those of the authors and do not necessarily represent the official position of the US Centers for Disease Control and Prevention.
